# Cerebrospinal Fluid Levels of Amyloid Precursor Protein Are Associated with Ventricular Size in Post-Hemorrhagic Hydrocephalus of Prematurity

**DOI:** 10.1371/journal.pone.0115045

**Published:** 2015-03-04

**Authors:** Diego M. Morales, Richard Holubkov, Terri E. Inder, Haejun C. Ahn, Deanna Mercer, Rakesh Rao, James P. McAllister, David M. Holtzman, David D. Limbrick

**Affiliations:** 1 Department of Neurological Surgery, Washington University in St. Louis, School of Medicine, Saint Louis, Missouri, United States of America; 2 Department of Pediatrics, University of Utah School of Medicine, Salt Lake City, Utah, United States of America; 3 Department of Pediatrics, Washington University in St. Louis, School of Medicine, Saint Louis, Missouri, United States of America; 4 Department of Newborn Medicine, Washington University in St. Louis, School of Medicine, Saint Louis, Missouri, United States of America; 5 Department of Neurology, Washington University in St. Louis, School of Medicine, Saint Louis, Missouri, United States of America; 6 The Hope Center for Neurological Disorders, Washington University in St. Louis, School of Medicine, Saint Louis, Missouri, United States of America; The University of Melbourne, AUSTRALIA

## Abstract

**Background:**

Neurological outcomes of preterm infants with post-hemorrhagic hydrocephalus (PHH) remain among the worst in infancy, yet there remain few instruments to inform the treatment of PHH. We previously observed PHH-associated elevations in cerebrospinal fluid (CSF) amyloid precursor protein (APP), neural cell adhesion molecule-L1 (L1CAM), neural cell adhesion molecule-1 (NCAM-1), and other protein mediators of neurodevelopment.

**Objective:**

The objective of this study was to examine the association of CSF APP, L1CAM, and NCAM-1 with ventricular size as an early step toward developing CSF markers of PHH.

**Methods:**

CSF levels of APP, L1CAM, NCAM-1, and total protein (TP) were measured in 12 preterm infants undergoing PHH treatment. Ventricular size was determined using cranial ultrasounds. The relationships between CSF APP, L1CAM, and NCAM-1, occipitofrontal circumference (OFC), volume of CSF removed, and ventricular size were examined using correlation and regression analyses.

**Results:**

CSF levels of APP, L1CAM, and NCAM-1 but not TP paralleled treatment-related changes in ventricular size. CSF APP demonstrated the strongest association with ventricular size, estimated by frontal-occipital horn ratio (FOR) (Pearson R = 0.76, p = 0.004), followed by NCAM-1 (R = 0.66, p = 0.02) and L1CAM (R = 0.57,p = 0.055). TP was not correlated with FOR (R = 0.02, p = 0.95).

**Conclusions:**

Herein, we report the novel observation that CSF APP shows a robust association with ventricular size in preterm infants treated for PHH. The results from this study suggest that CSF APP and related proteins at once hold promise as biomarkers of PHH and provide insight into the neurological consequences of PHH in the preterm infant.

## Introduction

Intraventricular hemorrhage (IVH) is the most common, severe neurological complication of preterm birth, occurring in roughly 25% of very low birth weight infants[1]. Post-hemorrhagic hydrocephalus (PHH) occurs in up to one half of those with IVH [2] and is associated with a 3–4 fold increase in the risk of cognitive and psychomotor disability [3]. Infants with PHH who require ventriculoperitoneal shunts (VPS) suffer the worst neurological outcomes, however, with neurodevelopmental impairments observed in >85% of extremely low birth weight infants and cerebral palsy in nearly 70%[4].

Despite the profound morbidity associated with PHH, there remain few clinical, radiographic, or laboratory parameters to guide treatment for PHH. Physical signs such as occipitofrontal circumference (OFC, or head circumference), splaying of the cranial sutures, and tenseness of the anterior fontanel are imprecise measures, and changes in vital signs such as apnea or bradycardia occur only late in the disease course. Imaging-based measures of ventricular size are frequently used for individualized treatment; yet ventricular size and/or morphology may be affected by IVH, hypoxia-ischemia, white matter injury, and impaired brain development—all of which are common among preterm infants[5]. Thus, there is a need to develop new tools to complement ventricular measures and inform the treatment of PHH.

Using tandem multi-affinity immunodepletion quantitative nano-LC-MS proteomics, we previously observed alterations in the abundance of key protein mediators of neurodevelopment in the CSF of a different group of infants with PHH [6] prior to ventricular decompression. A cohort of these proteins, including amyloid precursor protein (APP), neural cell adhesion molecule-L1 (L1CAM), and neural cell adhesion molecule-1 (NCAM-1), returned to control levels after neurosurgical decompression was initiated [6]. APP has been shown to play a role in synapse formation and repair, and neural plasticity [7,8]. Cleavage of APP may lead to amyloid beta, which is the primary component of amyloid plaques frequently found in the brains of patients with Alzheimer’s Disease [9–11]. L1CAM is a cell adhesion molecule that generates transmembrane signals via tyrosine kinase receptors [12–15] and plays multiple roles through development, such as neuronal migration, axonal growth, and synaptogenesis [15–17]. Mutations in L1CAM have been linked to MASA syndrome, X-linked hydrocephalus, and CRASH syndrome [18–22]. NCAM-1 is a transmembrane protein critical for cell migration, cell survival, axonal guidance, and synaptic targeting and plasticity associated with cognitive function [23–27]. In the current study, we measured serial CSF APP, L1CAM, and NCAM-1 concentrations in prematurely born infants throughout the interval when neurosurgical treatment of PHH was carried out; specifically, we examined the relationship of CSF APP, L1CAM, and NCAM-1 to ventricular size, total CSF protein, occipitofrontal circumference (OFC), and volume of CSF removed as part of clinical PHH treatment. Herein, we report the novel observation that CSF levels of APP are associated with ventricular size and may hold promise as a candidate biomarker of PHH but also provide insight into the neurological sequelae of PHH.

## Materials and Methods

### Ethics Statement

Approval from the Washington University Human Research Protection Office (WU-HRPO) was acquired prior to initiation of this study. Informed consent was obtained from subjects’ parent(s) or guardian(s) in all cases. As enrollment, consent, and/or sample acquisition were frequently performed while the mother was receiving medical care at another facility or was unable to travel to St. Louis Children’s Hospital, and as the study posed no more than minimal risk to participants, verbal consent was deemed sufficient by the WU-HRPO (#201101887). In the case of control subjects, written informed consent was obtained (WU-HRPO #201107082, 202013004). Consent, verbal or written, was documented in a regulatory binder for each participant.

### Research Subjects

As part of routine clinical care, all preterm infants ≤34 weeks estimated post-menstrual age (PMA) admitted to the St. Louis Children’s Hospital Neonatal Intensive Care Unit (NICU) underwent routine head ultrasound (US) examinations between 0–5 days of age to screen for IVH. Those found to have IVH then had weekly ultrasounds to monitor for PHH, and their OFC was recorded daily. Infants who demonstrated a frontal-occipital horn ratio (FOR[28]) ≥0.55 and progressive increase in OFC, full fontanel (palpated above the level of surrounding bone), and splaying of the sagittal suture ≥2 mm in the mid-parietal region[29] underwent implantation of a ventricular reservoir (RES) to treat PHH. Once a RES was implanted, semi-quantitative assessment of ventricular size and FOR (measured on head US performed 1–2 times/week), combined with clinical parameters (OFC, fontanel tenseness, and suture splaying), were used to direct the timing, frequency, and volume (typically 10–20 ml/kg per RES tap) of CSF removal for ventricular decompression. At term-equivalent age (TEA, 37^0/7^–41^6/7^ weeks PMA), the requirement for continued CSF removal was assessed, and a determination was made if the infant required a permanent ventriculoperitoneal shunt (VPS).

The current study spanned 2.5yrs, during which time 25 infants underwent implantation of a RES for PHH. Consent, verbal or written (see *Ethics Statement* above), was obtained in all cases; however, only the 12 subjects included herein had ≥5 ‘paired’ CSF samples and ultrasound examinations, where both the CSF sample and the ultrasound were acquired within a 24 window, an *a priori* criterion for enrollment in the current study. Eleven of the 12 subjects required a VPS; the remaining subject was transitioned to palliative care status due to cardiopulmonary disease and expired prior to TEA. In those who underwent VPS, the final CSF sample was obtained at the time of shunt implantation. None of the subjects enrolled in the study developed CSF, RES, or VPS infections. In order to account for differences in clinical risk severity uniformly across the cohort, subjects were scored using the Clinical Risk Index for Babies II (CRIB II)[30] taking into account PMA, sex, birthweight, and base excess. As temperature at admission has been shown to be heavily influenced by patient care itself, subject clinical risk index scores were reported using the CRIB II-T system [31]. Length of ventilation (LOV) was also recorded as a second established metric of severity of illness in preterm neonates [32].

### Ultrasound Measurements of Ventricular Size

Cranial ultrasound examinations (US) examinations were obtained in infants with PHH at least 1–2 times per week using a Zonarez.one UltraSmartCart diagnostic ultrasound system (Mountain View, CA). Ventricular size was measured on each US using two previously reported methods: ventricular index (VI; while bilateral, the larger of the two VI values was used)[33] and frontal-occipital horn ratio (FOR)[28]. No US was performed beyond what was required for clinical purposes. As the intent of this study was to evaluate the association between CSF proteins and ventricular size, only those US obtained within 24 hours of a CSF sample were used for analysis.

### Cerebrospinal Fluid Collection

CSF samples were obtained either directly from the operating room at the time of initial ventricular cannulation (during reservoir implantation) or at the NICU bedside at the time of percutaneous ventricular or RES taps. Final CSF samples were collected from the operating room during VPS surgery. In all cases, CSF was acquired under sterile conditions and only as clinically indicated. Once acquired from the operating room or NICU, each CSF sample was transported to the Washington University Neonatal CSF Repository on ice and then centrifuged at 2500 rpm for 6 minutes. The supernatant was then aliquotted into microcentrifuge tubes (500μl) and stored at -80°C until experimental analysis.

### Plasma Collection

Cord blood was collected at term delivery from healthy newborn infants (in evacuated tubes with anticoagulant), plasma separated within 2 hours, aliquotted and stored at -80°C until analysis.

### APP, L1CAM, and NCAM-1 Concentrations

Enzyme-Linked ImmunoSorbent Assays (ELISAs) were used to examine the CSF concentrations of APP, L1CAM, and NCAM-1 throughout the PHH treatment interval. Sandwich DuoSet ELISA development systems (R&D Systems, catalog #DY-850 and DY-2408, Minneapolis, MN) were used to measure APP and NCAM-1, as previously described [6]. Briefly, high-binding polystyrene microplates (Costar, Corning Life Sciences, INC., Lowell, MA) were coated with mouse monoclonal anti-human APP or NCAM-1 primary antibody (4μg/mL and 2μg/mL respectively). The plate was then blocked for an hour in 1% BSA. The CSF samples and standards were incubated and then biotinylated goat anti-human APP or NCAM-1 secondary antibody was added (300ng/mL and 200ng/mL respectively) followed by a streptavidin-HRP reagent. After, the working stabilized hydrogen peroxide solution and stabilized tetramethylbenzidine were loaded and incubated prior to the addition of sulfuric acid. L1CAM concentrations were measured using a commercially available ELISA kit (DRG, catalog #EIA5074, Mountainside, NJ) following the manufacturers protocol, also as previously described[6]. ELISAs were run in duplicate, and the 96-well plates were read at 450nm on a Versamax microplate reader (Molecular Devices, Sunnyvale, CA). Actual APP, L1CAM, and NCAM-1 concentrations were determined using a four parameter logistic standard curve as detailed by the manufacturers.

### Total Protein Measurements

The Pierce Bicinchoninic Acid (BCA) protein assay kit (Thermo Scientific, Waltham, MA) was used to determine the total concentration of protein in each CSF sample. Briefly, 25μl of CSF and BSA standards were placed into the microplate wells (in duplicates); 200μl of the working reagent was then added and the plate was incubated for 30min at 37°C. The plate was cooled to room temperature and the absorbance was measured at 562nm on a plate reader. Total protein concentrations were determined using a four parameter logistic standard curve.

### Statistical Analysis

Data were expressed using mean ± standard deviation. Associations between continuous factors were assessed using the Pearson correlation coefficient. Rates of linear change over time for markers and other measures were calculated using standard linear regression with the marker/factor as outcome and timepoint as predictor. All data analyses were performed using SAS 9.2.

## Results

### Patient Characteristics and PHH Treatment

Twelve prematurely born subjects (7 male, 5 female) with gestational age of 25.7±2.5 weeks and birthweight of 865±361 grams were included. Characteristics of the cohort are shown in Table 1. IVH was diagnosed prior to 31 weeks PMA (26.3±2.4 weeks) in all patients. RES and VPS implantation were performed at 30±4 weeks (range 25–38) and 38±2 weeks (range 34.6–40.9), respectively. The mean frequency for CSF removal and mean volume of CSF withdrawn per procedure were 3±2 times/week and 13.9±4.9ml, respectively.

**Table 1:** Characteristics of the 12 subjects with post-hemorrhagic hydrocephalus enrolled in the study.

Subject Number	Race	Sex	Birth weight (g)	Birth PMA (wks)	CRIBII-T	# of US	# of CSF samples	Average CSF removed (ml)	IVH Dx EGA (wks)	Reservoir Surgery EGA (wks)	Shunt Surgery EGA (wks)
1	Black	F	620	24 4/7	13	6	10	12.63	24 5/7	27 6/7	37 6/7
2	Caucasian	M	650	25 5/7	13	4	6	15.00	28 2/7	37 5/7	40 5/7
3	Black	M	740	24 3/7	14	23	46	11.88	24 5/7	28 4/7	37 3/7
4	Black	F	740	24	14	7	11	13.63	24 2/7	27 6/7	34 5/7
5	Caucasian	M	835	29	10	9	17	13.70	29	31 4/7	40 6/7
6	Caucasian	M	850	25 2/7	12	14	24	18.31	25 3/7	28	40
7	Asian	M	700	24	15	15	57	15.08	25 6/7	29 2/7	39 1/7
8	Black	M	750	24	14	23	53	9.01	24 2/7	27	37 4/7
9	Caucasian	F	730	23 6/7	13	6	12	8.82	24 1/7	28 4/7	N/A
10	Black	F	610	23 1/7	14	12	26	6.67	24 3/7	25	37
11	Caucasian	F	1300	29 4/7	3	14	34	17.80	29 5/7	31 2/7	36 4/7
12	Caucasian	M	1854	30 4/7	3	5	13	25.25	30 5/7	36 3/7	40 3/7

### Cerebrospinal Fluid Protein and APP, L1CAM, and NCAM-1 Measurements

**Fig 1:**
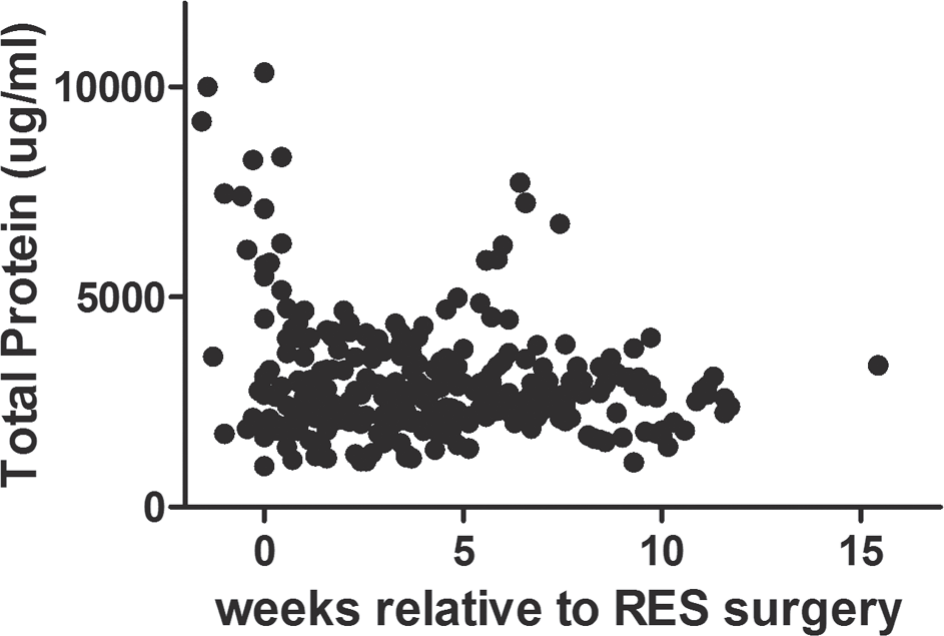
Total protein. Total protein measurements (μg/ml) for all 12 PHH subjects analyzed throughout the duration of the study.

Variability was observed in the total protein (TP) in the CSF of the 12 PHH subjects, both in terms of absolute levels and dynamic trends in concentration (increases/decreases) throughout the PHH treatment interval (Fig. 1). Initial TP level was not associated with CRIB II-T (R = 0.24, p = 0.45) or LOV (R = -0.02, p = 0.96), proxies for critical illness in preterm infants, or with length of time from IVH diagnosis to acquisition of the CSF sample (R = 0.03, p = 0.91). Trends in TP tracked independently of changes in measured APP (R = -0.13, p = 0.69) and showed marginal correlations with CSF L1CAM (R = -0.34, p = 0.28) and NCAM-1 (R = -0.45, p = 0.14), respectively. Thus, in order to facilitate across-subject analyses, the CSF levels of these proteins were normalized by TP elsewhere in this study, except where noted.

### Cerebrospinal Fluid APP, L1CAM, and NCAM-1 Levels, and Ventricular Size Metrics

In order to evaluate the relationship between candidate CSF biomarkers and ventricular size, two commonly referenced ultrasound-based ventricular size parameters were assessed for strength of association with CSF APP, L1CAM, and NCAM-1 levels. Specifically, ventricular index (VI, measured bilaterally, the larger of the two was chosen for analysis) and FOR were analyzed. FOR, which is ratiometric and takes into account the bilateral frontal and occipital horns of the lateral ventricles and the brain size, consistently showed a more robust relationship to normalized CSF levels of APP, L1CAM, and NCAM-1 than VI (Table 2). Thus, FOR was selected as the primary ventricular measurement to report the association of these candidate CSF proteins to ventricular size. Of note, CSF TP alone did not demonstrate an appreciable correlation with FOR (R = 0.02, p = 0.95).

**Table 2:** The Pearson Correlation Coefficients (and p-value) for normalized proteins and their correlation with the ventricular size metrics Ventricular Index (VI) and Frontal-Occipital Horn Ratio (FOR).

Protein*	VI	FOR
APP	0.58 (0.047)	0.76 (0.004)
L1CAM	0.51 (0.087)	0.57 (0.055)
NCAM-1	0.57 (0.051)	0.66 (0.019)

* All levels normalized by total protein.

### Cerebrospinal Fluid APP, L1CAM, and NCAM-1, and Ventricular Size throughout the PHH Treatment Period

**Fig 2:**
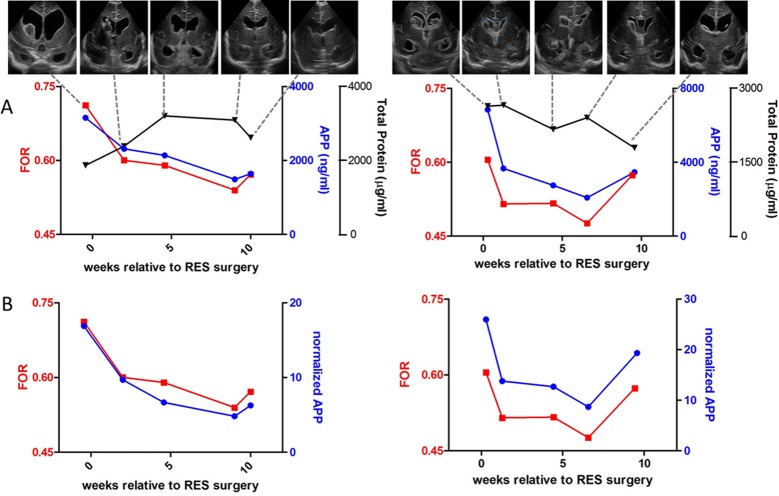
FOR and APP levels. **A**.FOR measurements (red left y-axis) and absolute APP concentrations (blue right y-axis) and total protein (black right y-axis) plotted versus relative time of reservoir surgery in selected timepoints of patients #7 (left) and #8 (right). Each FOR data point is accompanied by an US image for the selected patient at that particular timepoint. The image shown is a coronal slice taken at the foramen of Monro. **B**. FOR measurements (left y-axis) and normalized APP levels (right y-axis) plotted versus relative time of reservoir surgery in selected timepoints of patients #7 (left) and #8 (right).

As detailed above, CSF was removed via RES taps as clinically indicated for the treatment of each infant. To evaluate the relationship between CSF APP, L1CAM, and NCAM-1 levels and ventricular size, the levels of each protein were measured in CSF samples obtained within 24h of each cranial US. Fig. 2 shows the relationship between CSF APP levels and FOR for two typical subjects (data for all other subjects are included in S1–S7 Figs.). Fig. 2A includes TP and absolute APP values, and Fig. 2B shows APP levels normalized by TP. Actual clinical ultrasound images are provided in the insets for reference. A robust correlation was observed between CSF APP and FOR, with CSF APP tracking ventricular size closely throughout the PHH treatment interval.

**Fig 3:**
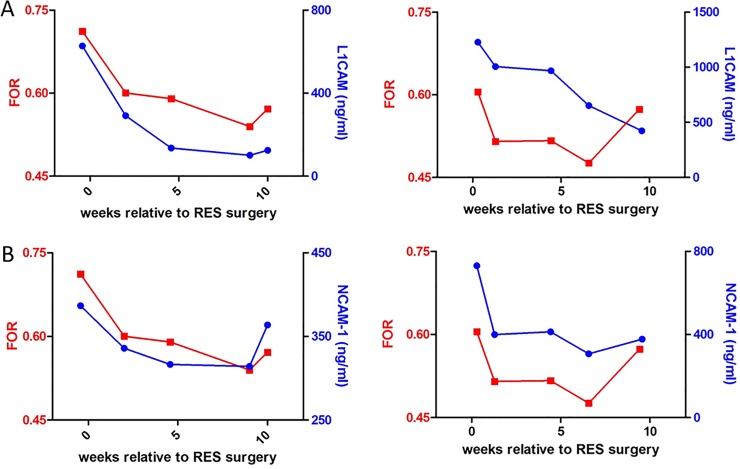
FOR, L1CAM, and NCAM-1 levels. **A**.FOR measurements (red left y-axis) and absolute L1CAM concentrations (blue right y-axis) plotted versus relative time of reservoir surgery in selected timepoints of patients #7 (left) and #8 (right). **B**. FOR measurements (left y-axis) and absolute NCAM-1 concentrations plotted versus relative time of reservoir surgery in selected timepoints of patients #7 (left) and #8 (right).

Common neurosurgical practice is to taper or stop RES taps prior to VPS implantation in order to: 1) assess the need for permanent VPS after temporizing PHH treatment with RES taps (i.e. to evaluate each subject’s ability to regain the capacity to absorb CSF); and 2) facilitate the function of the VPS, if one is required (to “prime” the VPS). As shown in Fig. 2, CSF APP detected the expected increase in ventricular size as RES taps were stopped. Similar observations were made with CSF L1CAM (Fig. 3A) and CSF NCAM-1 (Fig. 3B).

**Fig 4:**
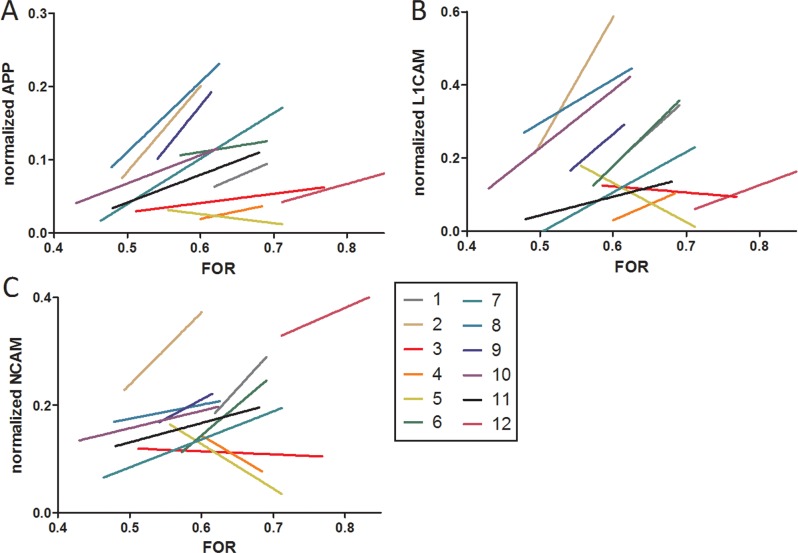
Correlation of FOR with normalized proteins. A linear regression analysis showing the correlation of normalized APP (A), normalized L1CAM (B), and normalized NCAM-1 (C) with FOR measurements for all 12 subjects used.

In order to quantitate the strength of association between normalized CSF APP, L1CAM, and NCAM-1 with FOR, the rate of linear change in each CSF protein was plotted against the rate of linear change in FOR for each subject (Fig. 4A-C). While there was some variability in the rate of linear change for each CSF protein among subjects, likely due to inherent variation in the treatment of individual subjects, a strong correlation was observed for change in FOR with changes in CSF APP (R = 0.76, p = 0.004); a moderately strong correlation was also observed for CSF NCAM-1(R = 0.66,p = 0.02), and a trend toward correlation was seen with CSF L1CAM(R = 0.57,p = 0.055) (Table 2). Of note, there was no relationship between FOR and TP (R = 0.02, p = 0.95), and the associations between FOR and OFC (R = 0.36, p = 0.25), and volume of CSF removed (R = -0.13, p = 0.70) were very weak.

### Initial Peak APP, L1CAM, and NCAM-1 Levels, and Ventricular Size

In addition to examining the relationship between APP, L1CAM, and NCAM-1 levels and ventricular size in individual infants in repeated CSF samples over time, we also examined the CSF levels of these proteins across all subjects at the time of the initial CSF sample (at the start of temporizing PHH treatment, i.e. previously untreated PHH). After normalizing for TP, APP, L1CAM, and NCAM-1 levels, measured in CSF obtained at the time of initiation of PHH treatment, were not consistently associated with FOR (R = -0.11, -0.14, and 0.17, respectively). Similarly, no clear association was observed between initial APP, L1CAM, or NCAM-1 levels and length of time (in days) after IVH diagnosis (R = 0.18, -0.23, and 0.09 respectively).

### Plasma Levels of APP, L1CAM, and NCAM-1

As IVH is a critical factor in the pathogenesis of PHH, it is important to exclude blood itself as a major potential source for CSF APP, L1CAM, and NCAM-1. To this end, we examined the concentration of APP, L1CAM, and NCAM-1 in the plasma of age-matched infants without PHH or other known neurological injury. In this cohort, plasma APP = 126±51ng/ml, plasma L1CAM = 13±5ng/ml, and plasma NCAM-1 = 442±102ng/ml. For comparison, the concentrations of APP, L1CAM, and NCAM-1 measured in the initial CSF samples obtained in the 12 PHH subjects included in this study were 3194±1952ng/ml, 771±472ng/ml, and 483±183ng/ml, respectively. In contrast to NCAM-1 (p = 0.53), APP and L1CAM demonstrated significantly higher levels in PHH CSF than in the plasma of age-matched infants without PHH or other neurological injury (p = 0.0003 and p = 0.001, respectively).

## Discussion

The current study was directed at evaluating the relationship between CSF levels of APP, L1CAM, and NCAM-1, with ventricular size. Our previous work suggested that these and other specific protein mediators of neurodevelopment exhibited increased levels in the CSF of infants with untreated PHH, but they decreased to control levels after the initiation of neurosurgical treatment[6]. Using tandem multi-affinity proteomics, we previously identified a cohort of CSF proteins, including APP, L1CAM, and NCAM-1, that demonstrated marked differences between PHH and control. In the current study, we observed that the CSF levels of APP paralleled changes in ventricular size throughout the clinical PHH treatment interval. We also noted a trend toward moderate association between L1CAM and ventricular size which did not achieve statistical significance likely related to the small sample size. NCAM-1 was correlated with ventricular size, though to a lesser extent than APP, but was not enriched in PHH CSF. Taken together, our results suggest that APP, and possibly other related proteins, hold promise as biomarkers of PHH, potentially complementing physical parameters and existing image-based measures of ventricular size in the treatment of this condition.

The finding that CSF proteins, particularly APP or others with involvement in neurodevelopmental processes may be associated with ventricular size lends support to the notion that these proteins may represent candidate markers of PHH and its associated neurological disability. At present, crude physical findings and semi-quantitative parameters of ventricular size, often obtained in the setting of abnormal ventricular morphology (resulting from adjacent white matter injury/loss, porencephaly, or other causes) are used to direct treatment for PHH. Thus, there is significant momentum to develop novel candidate PHH biomarker to complement existing physical and image-based parameters to aid in the diagnosis and management of PHH.

The mechanism by which PHH results in increases in the CSF levels of APP, L1CAM, NCAM-1, and perhaps other proteins remains unclear. While it would seem possible that the initial IVH and blood products that remain in the CSF could result in elevations in these CSF proteins (or total CSF protein more generally), the data reported herein do not support this explanation. Using age-matched control plasma samples, we found that APP and L1CAM levels are 25 fold and 59 fold (respectively) higher in PHH CSF than control plasma, making it unlikely that blood products alone could be responsible (the notable exception was NCAM-1, where there was no difference between control plasma and PHH CSF).

In the current study, CSF APP tracked ventricular size closely, even demonstrating increases when ventricular size increased. These findings also argue against blood products as the origin of these CSF protein levels; rather, they raise the possibility that secondary axonal and/or neuronal injury may be contributing to elevations. Amyloid precursor protein is known to be released by axonal injury or stretch[34]; thus it is plausible that ventricular distention in hydrocephalus, and axonal stretch as a consequence, could represent the origin of the observed changes in CSF APP.

In the setting of hydrocephalus, increased ventricular size may be associated with increased intracranial pressure (ICP). While it is of interest to determine the relationship of these candidates CSF biomarkers to ICP, this presents several challenges in the study of human preterm infants with PHH. First, the validity of ICP measurements in preterm infants with patent cranial sutures is unclear, since the open sutures likely attenuate increases in ICP. For this reason OFC is often used as a surrogate for ICP, but this measurement is subject to measurement error under the best of conditions. Further, treatment of PHH with RES is dominated by intermittent CSF removal, leading to fluctuations in ICP, rather than the more continuous CSF diversion provided through ventriculo-subgaleal shunts or VPS; however, by their very nature (risk of infection), routine CSF access is not practical with these devices. Further, ICP in the preterm infant with PHH may be affected by a number of other factors independent of CSF removal, including IV sedation, anesthesia (particularly at surgery), and wide variations in ventilator support, all of which would be expected to influence ICP. Finally and most importantly, bedside percutaneous RES taps are often accompanied by crying and movement by the infant, which inevitably transiently increases ICP during the tap, complicating the use of traditional manometers and the interpretation of their values. However, we were able to record ICP (measured as opening pressure on manometry after insertion of the needle into the RES) in two infants with serial RES sampling. While preliminary, ICP and normalized APP did demonstrate an association in these two infants (R = 0.59 and 0.57, respectively). Currently, we are investigating CSF APP, L1CAM, and other proteins in hydrocephalus of varying etiologies that affect older children and adolescents, where many of these issues will be mitigated, permitting a clearer assessment of the relationship between candidate CSF markers, ICP, and ventricular size. However, it is likely that animal models will ultimately be needed to fully resolve this issue.

The findings of this study are subject to a number of limitations. The data were derived from a small number of preterm infants (n = 12), with inherent variability in medical co-morbidities and clinical care. In addition, 11 of the 12 subjects required a permanent VPS, while the final subject expired from non-neurological causes before reaching TEA, the point at which VPS is considered. Since only 70–80% of patients typically require VPS[35,36], it is possible that our sample of 12 patients represents a biased sample; certainly, by excluding patients that did not require serial CSF removal, the VPS rate in this study would be expected to be higher than studies that report VPS rates for all infants treated for post-hemorrhagic ventricular dilatation. Alternatively, it could be argued that each subject, by requiring VPS, had confirmed PHH, rather than simply post-hemorrhagic ventricular dilatation. Regardless, the findings reported herein must be confirmed in a larger sample size. To this end, a multi-institutional study of candidate CSF markers of PHH is currently underway through the Hydrocephalus Clinical Research Network (HCRN). Finally, the scope of this study was limited to CSF APP, L1CAM, and NCAM-1; as demonstrated previously[6], there are a number of important proteins (e.g. amyloid isoforms, tau, others) to be assessed for association with ventricular size.

## Conclusion

In the current study, we report the novel observation that the CSF level of APP is associated with ventricular size in preterm infants undergoing treatment for PHH. The results from this study suggest that specific CSF proteins, such as APP, when paired with physical parameters and traditional imaging-based measures of ventricular size, may provide additional information regarding disease status and therapeutic efficacy. Future work will be directed at evaluating the role of CSF APP and related proteins as candidate markers of PHH and at investigating the pathophysiological implications and developmental consequences of these CSF protein changes.

## Supporting Information

S1 Fig Total CSF protein and FOR. Total CSF protein concentrations (μg/mL) (blue left y-axis) and FOR measurements (red right y-axis) plotted versus relative time of reservoir surgery in all timepoints used for all twelve subjects.
(TIF)

S2 Fig CSF APP concentrations and FOR. CSF Amyloid Precursor Protein concentrations (ng/mL) (blue left y-axis) and FOR measurements (red right y-axis) plotted versus relative time of reservoir surgery in all timepoints used for all twelve subjects.
(TIF)

S3 Fig CSF L1CAM concentrations and FOR. CSF L1CAM concentrations (ng/mL) (blue left y-axis) and FOR measurements (red right y-axis) plotted versus relative time of reservoir surgery in all timepoints used for all twelve subjects.
(TIF)

S4 Fig CSF NCAM-1 concentrations and FOR. CSF NCAM-1 concentrations (ng/mL) (blue left y-axis) and FOR measurements (red right y-axis) plotted versus relative time of reservoir surgery in all timepoints used for all twelve subjects.
(TIF)

S5 Fig Normalized CSF APP and FOR. Normalized CSF Amyloid Precursor Protein levels (blue left y-axis) and FOR measurements (red right y-axis) plotted versus relative time of reservoir surgery in all timepoints used for all twelve subjects.
(TIF)

S6 Fig Normalized CSF L1CAM and FOR. Normalized CSF L1CAM levels (blue left y-axis) and FOR measurements (red right y-axis) plotted versus relative time of reservoir surgery in all timepoints used for all twelve subjects.
(TIF)

S7 Fig Normalized CSF NCAM-1 and FOR. Normalized CSF NCAM-1 levels (blue left y-axis) and FOR measurements (red right y-axis) plotted versus relative time of reservoir surgery in all timepoints used for all twelve subjects.
(TIF)
